# Association between multidimensional frailty and quality of life in older adults with coronary heart disease: a cross-sectional study

**DOI:** 10.3389/fpsyg.2025.1563493

**Published:** 2025-05-09

**Authors:** Dongmei Mei, Yan Yang, Defang Meng, Yaoy Hu, Xiaoyan Wang

**Affiliations:** ^1^Medical College of Jiangnan University, Wuxi, China; ^2^Affiliated Hospital of Jiangnan University, Wuxi, China

**Keywords:** multidimensional, frailty, quality of life, older adults, coronary heart disease

## Abstract

**Purpose:**

This study aims to investigate the relationship between multidimensional frailty and quality of life among older adults with coronary heart disease who are hospitalized.

**Patients and methods:**

A convenience sampling approach was employed to select older adults with coronary heart disease admitted to the Cardiology Department of a tertiary hospital in Wuxi City between September 2023 and June 2024. The study utilized the General Information Questionnaire, the Tilburg Frailty Indicator (TFI), and the SF-12 Scale to gather data. To examine the relationship between multidimensional frailty and quality of life, Spearman correlation analysis was conducted. Additionally, stratified regression analysis was performed to identify factors associated with quality of life.

**Results:**

Among 254 older adults with coronary heart disease, 135 (53.15%) exhibited multidimensional frailty. The physical health score of frail older patients with coronary heart disease (36.62 ± 6.51) was found to be lower than that of their non-frail counterparts (42.88 ± 6.91). A negative correlation was observed between multidimensional frailty and quality of life, encompassing both physical and mental health aspects (*r* = −0.513, −0.428, *p* < 0.01). Further analysis using stratified regression revealed that multidimensional frailty independently accounted for 9.1 and 11.3% of the variance in quality of life (physical health and mental health, respectively) among older adults with coronary heart disease.

**Conclusion:**

Older adults with coronary heart disease often experience widespread frailty, which is linked to their quality of life. The presence of multifaceted weakness serves as a crucial indicator of life quality in these individuals.

## 1 Introduction

The demographic composition of society has changed significantly. There is a notable increase in the aging population and a rise in chronic disease prevalence. Cardiovascular diseases have seen a continuous uptick in morbidity and mortality rates, with projections indicating that 330 million individuals in China are affected by these conditions. Among them, the prevalence rate of CHD among older adults in China is approximately 27.8 ‰ aged 60 years and over (Wang, 2023), 11.4 million people are battling coronary heart disease (CHD) (Diseases NCfC, Health TWCotRoC, China Di, 2024) which stands as the primary cause of heart disease-related deaths and negatively impacts the older adult’s quality of life. Research has indicated that various factors, including socio-demographic characteristics, lifestyle choices, and psychological well-being, play a role in influencing the quality of life experienced by older adults with CHD (Chu et al., 2019; Miao et al., 2019; Liu et al., 2023).

As the population ages, frailty has emerged as a common geriatric condition. A systematic review and meta-analysis of 64 studies involving 106,826 participants from 23 provinces in China reported that the prevalence of frailty among community-dwelling older adults was approximately 10.1% (95% CI: 8.5–11.7%) (Zhou et al., 2023). Research indicates that frailty affects 30% of older patients with CHD (Xiamin et al., 2023). Frailty older CHD adults face higher risks of unplanned readmission and all-cause mortality compared to their non-frail counterparts, with rates of 36.1 and 11.4%, respectively (Ning et al., 2021). Frailty status serves as a crucial indicator of adverse cardiovascular events and mortality in older CHD patients (Ning et al., 2021), significantly impacting patient outcomes and life quality. While existing studies have explored the relationship between frailty and both prognosis and quality of life, research specifically examining multidimensional frailty—including physical, cognitive, psychological, and social aspects—and its impact on quality of life in older patients with CHD, particularly within tertiary care settings, remains limited. A study focusing on hospitalized cardiac patients revealed a general decline in quality of life and an increase in psychological issues among these individuals (Tang et al., 2021). Unlike previous studies that predominantly addressed physical frailty, multidimensional frailty encompasses a broader range of factors. For instance, a five-year longitudinal study demonstrated that multidimensional frailty, assessed using the Multidimensional Prognostic Index (MPI), more accurately predicts mortality in community-dwelling older adults compared to physical frailty assessments like the Cardiovascular Health Study (CHS) index (Cella et al., 2021). Given the increasing prevalence of frailty in older CHD patients, understanding its multidimensional. Gobbens et al. (2015) introduced the concept of multidimensional frailty, proposing an integrated model where various factors contribute to a dynamic state of decline in one or more domains (physical, psychological, social), increasing the likelihood of negative outcomes. This integrated approach to frailty emphasizes treatment and prevention strategies, incorporating holistic care concepts and interdisciplinary collaboration. This aligns with the current ‘biopsychosocial’ model in modern medicine and resonates with China’s promotion of healthy aging and wellness (Yuhui et al., 2024). Consequently, an increasing number of researchers are exploring the multidimensional nature of frailty (Xue et al., 2024), highlighting its complexity in older adults and advocating for a comprehensive management approach. This emphasis on frailty’s impact on quality of life and prognosis in older CHD adults aims to raise awareness among clinical staff and ultimately enhance patient well-being.

The global population has experienced significant aging in recent times, prompting the World Health Organization to introduce the concept of active aging as a means to address this worldwide trend (Abdullah and Wolbring, 2013). Aging is a natural stage of life that entails physical, psychological, and social changes. As people age, it is important to take measures to maintain or improve their quality of life in order to age actively and healthily. Among the most recommended non-pharmacological strategies for improving quality of life in old age is physical exercise (Parra-Rizo, 2020). Strength training is an effective intervention. It not only improves muscle strength and balance but also helps reduce fall frequency, improves overall physical condition, and decreases the fear of falling, which together improve the quality of life and autonomy of older adults (Rivera Miranda et al., 2024). Evidence suggests that active aging plays a pivotal role in safeguarding the physical and mental health of older adults, ensuring their quality of life, and slowing down the progression of frailty (Maidan et al., 2023). Currently, most studies concentrate on the physical frailty status and quality of life of older CHD adults, which does not align with the principles of active aging and the contemporary biopsychosocial medical model. Consequently, it is essential to examine the multidimensional frailty condition of older adults and its effects on their quality of life. This approach seeks to enhance clinical recognition of frailty’s multidimensional aspects—physical, cognitive, psychological, and social—in hospitalized patients with CHD. It also aims to promote the implementation of targeted interventions by healthcare professionals. These interventions include structured exercise programmes, nutritional counseling, cognitive-behavioral strategies, and social support initiatives (Dent et al., 2019; Choi et al., 2023). By addressing the various dimensions of frailty, this method works to improve the overall quality of life for patients. This study investigated the relationship between multidimensional frailty and quality of life among older adults with CHD in Chinese hospitals. The goal was to provide guidance for clinical practice and contribute to improving patients’ health management. Building on this background, the following section outlines the methods employed to investigate multidimensional frailty in older adults with CHD.

## 2 Materials and methods

### 2.1 Research population

A study was conducted using convenience sampling to recruit older patients with CHD from the Cardiology Department of a tertiary hospital in Wuxi between September 2023 and June 2024. The research included 20 variables, as per observational research sample calculation guidelines. All potential participants were approached during their hospital stay and those who agreed to participate were interviewed face-to-face by trained researchers using a standardized questionnaire. Clinical data were also extracted from hospital records to ensure data accuracy. Prior to enrolment, written informed consent was obtained from each participant. According to the regression analysis, the sample size should be 5–10 times the number of independent variables plus an extra 10% to account for attrition. Aiming for a sample size of 112–223 people with 20 independent variables, of the 274 patients approached, 254 agreed to participate, a response rate of approximately 92.7% (Ping et al., 2010). The study ultimately included 254 cases. In order to enhance the robustness of the findings and to represent the population more fully. Participants were eligible if they: (1) had a confirmed clinical diagnosis of coronary heart disease: angiographically confirmed stenosis of ≥50% in ≥1 major coronary heart or previous revascularisation (according to ESC guidelines) (Byrne et al., 2023); (2) were 60 years or older; and (3) voluntarily agreed to participate, signed an informed consent form, and could communicate effectively. Exclusion criteria encompassed: (1) individuals with tuberculosis, advanced malignant tumors, HIV, severe chronic gastrointestinal diseases, or psychiatric disorders such as schizophrenia and depression; (2) those who had participated in a clinical drug trial within the past 3 months; and (3) patients classified as NYHA Cardiac Function Class IV. The Ethics Committee of the Affiliated Hospital of Jiangnan University approved this study (approval number LS2023085).

### 2.2 General information questionnaire

Following the researcher’s methodology, various patient data were extracted from medical records. These included demographic information such as age, gender, body mass index (BMI), and educational background. Additionally, lifestyle factors like living arrangements, treatment modalities, tobacco use, alcohol consumption, sleep patterns, and exercise habits were documented. The study also considered clinical aspects, including CHD classification, cardiac function status, presence of multiple chronic conditions (defined as two or more), and medication history.

### 2.3 The tilburg frailty indicator (TFI)

Gobbens et al. (2015) created the TFI in 2010 to evaluate multidimensional frailty in patients. The instrument consists of 15 items covering three domains: physical, psychological, and social frailty. Each item is scored as either ‘0’ or ‘1,’ with a maximum total score of 15 points. The physical frailty domain ranges from 0 to 8 points, psychological frailty from 0 to 4 points, and social frailty from 0 to 3 points. Multidimensional frailty is indicated by a score ≥5, while domain-specific frailty is indicated by scores ≥3 for physical frailty and ≥2 for both psychological and social domains. Higher scores indicate greater frailty severity (Gobbens et al., 2021). Xing et al. (2013) translated the scale into Chinese and validated it in older patients with chronic diseases. The Chinese version showed good reliability with a Cronbach’s *α* coefficient of 0.686 (Xing et al., 2013), making it a valid tool for assessing multidimensional frailty in older patients with chronic conditions in China.

### 2.4 The Q12-item short-form health survey (SF-12)

Ware et al. (1996) developed a simplified version called SF-12. This assessment consists of 12 items covering eight health dimensions: general health, physical functioning, physical role, emotional role, bodily pain, vitality, mental health, and social functioning. These dimensions are categorized into Physical Health (PCS) and Mental Health (MCS) components. The SF-12 requires data reassignment and standardized transformation of raw scores, utilizing specific assignment and transformation methods (Hongyu, 2016). Scores range from 0 to 100, with higher scores indicating better quality of life. The SF-12 has been extensively validated in a population of community-dwelling older adults in China, showing high internal consistency reliability (a Cronbach’s *α* coefficient of 0.910) (Shou et al., 2016) and good construct validity (the two-factor model explained 60.7% of the total variance) (Shou et al., 2016). It also showed strong convergent and discriminant validity, and correlated well with the SF-36 total score, making the scale suitable for Chinese older adults.

### 2.5 Survey and quality control methods

The study’s data was gathered through paper surveys administered within 48 h of hospital admission. The department’s head nurse received training content provided by the researcher and conducted the survey. The training covered the background, objectives and methods of the study, followed by several practical simulations. Nurses’ competence was assessed through pre-test assessments and ongoing performance monitoring during the pilot phase. Only after passing the competency assessment were they assigned to conduct the survey. In this study, only one investigator was employed to ensure consistency and quality of data. The researcher, who was also the survey administrator, provided participants with a comprehensive explanation of the study’s objectives and importance. Participants then gave their consent by signing an informed consent document before the survey began. Questionnaires were immediately collected upon completion. The retrieved forms were examined for completeness, with any incomplete surveys being discarded. The remaining questionnaires underwent a double-check process before the data was entered into the system.

A number of measures were taken to minimize the potential for bias. To address the issue of observer bias, all investigators were trained to collect data with a uniform terminology and to standardize data collection protocols. This included the use of standardized questionnaires and measurement tools, as well as blind testing of investigator-participant exposure where possible. This approach helped to reduce inter-observer variability and ensure consistency and objectivity in the assessment of results.

### 2.6 Statistical methods

Statistical analysis was conducted using SPSS27.0. For normally distributed quantitative data, results were presented as mean ± standard deviation ( $\bar{X}$ ±S) while skewed data were expressed as M (Q1, Q3). Qualitative data were reported as frequencies and percentages. One-way analyses employed t-tests and H-tests. The relationship between multidimensional frailty and quality of life was examined using Spearman correlation analysis. Stratified regression analyses were performed with quality of life scores as the outcome variable; and statistically significant factors from univariate analyses and multidimensional frailty as predictors. Model 1 incorporated general data as independent variables, while Model 2 added multidimensional frailty. Statistical significance was set at *p* < 0.05.

## 3 Results

### 3.1 Evaluating the quality of life metrics for older adults with CHD across various demographic and clinical factors

The study involved 254 older adults with CHD. Their average physical health score was 39.55 ± 7.39 points, with significant variations observed among groups based on age, exercise habits, sleep issues, cardiac function classification, and presence of multimorbidity groups (*p* < 0.05). The mean mental health score was 44.80 ± 4.98 points, showing significant differences between various sleep groups (*p* < 0.05). Further details are provided in Table 1.

**Table 1:** Assessment of life quality scores for older adults with CHD, categorized by various characteristics (*n* = 254).

Characteristic	Number	PCS	Statistic	*p*-value	MCS	Statistic	*p*-value
Age (years, $\bar{X}$ ±S)			*t* = 3.86	<0.001		*t* = 0.19	0.846
60–74	180 (70.87)	40.67 ± 7.13			44.84 ± 5.06		
≥75	74 (29.13)	36.83 ± 7.35			44.71 ± 4.82		
Sex			*t* = 1.71	0.088		*t* = 1.71	0.088
Male	175 (68.90)	40.08 ± 7.39			44.65 ± 4.83		
Female	79 (31.10)	38.37 ± 7.28			45.13 ± 5.31		
BMI [(kg/m^2^), $\bar{X}$ ±S]			*F* = 2.27	0.106		*F* = 0.85	0.427
<18.5	6 (2.36)	44.99 ± 9.23			43.97 ± 4.38		
18.5–23.9	102 (40.16)	38.80 ± 7.53			45.29 ± 5.30		
>23.9	146 (57.48)	39.85 ± 7.15			44.49 ± 4.77		
Education [ $\bar{X}$ ±S/M (Q1, Q3)]			*F* = 2.32	0.076		*H* = 4.51	0.211
Uneducated	16 (6.30)	38.34 ± 7.11			42.81 (40.85, 44.86)		
Primary	56 (22.05)	38.29 ± 7.70			45.40 (41.02, 48.34)		
Middle	119 (46.85)	40.84 ± 6.96			44.51 (41.47, 48.43)		
High school and above	63 (24.80)	38.55 ± 7.72			45.41 (42.61, 47.50)		
Marital status ( $\bar{X}$ ±S)			*F* = 2.45	0.089		*F* = 0.55	0.580
Unmarried	2 (0.79)	46.51 ± 5.87			42.69 ± 8.35		
Married	215 (84.65)	39.83 ± 7.23			44.71 ± 4.82		
Divorced or widowed	37 (14.57)	37.54 ± 8.01			45.47 ± 5.76		
Live alone ( $\bar{X}$ ±S)			*t* = −1.01	0.314		*t* = −0.01	0.995
Yes	22 (8.66)	41.07 ± 7.25			44.80 ± 4.87		
NO	232 (91.34)	39.41 ± 7.40			44.81 ± 6.19		
Medical insurance ( $\bar{X}$ ±S)			*F* = 0.55	0.651		*F* = 1.71	0.165
Countryside	21 (8.27)	37.93 ± 6.81			42.88 ± 4.57		
Urban residents	225 (88.58)	39.63 ± 7.49			45.02 ± 4.98		
Employee	6 (2.36)	41.24 ± 4.22			42.56 ± 5.60		
Other	2 (0.79)	42.41 ± 11.68			46.67 ± 2.72		
Types of CHD ( $\bar{X}$ ±S)			*t* = 0.78	0.439		*t* = −0.56	0.576
Acute type	142 (55.91)	39.87 ± 7.13			44.65 ± 4.99		
Stabilized	112 (44.09)	39.14 ± 7.71			45.00 ± 4.99		
Smoking ( $\bar{X}$ ±S)			*F* = 2.36	0.096		*F* = 0.43	0.649
Never	110 (43.31)	38.51 ± 7.76			44.91 ± 5.27		
Smoking	91 (35.83)	39.92 ± 6.95			44.44 ± 4.93		
Quit smoking	53 (20.87)	41.08 ± 7.10			45.19 ± 4.46		
Drinking alcohol ( $\bar{X}$ ±S)			*F* = 1.36	0.258		*F* = 0.17	0.847
Never	129 (50.79)	38.85 ± 7.32			44.95 ± 5.15		
Drinking	58 (22.83)	40.69 ± 7.05			44.49 ± 4.55		
Quit drinking	67 (26.38)	39.91 ± 7.75			44.79 ± 5.06		
Exercise ( $\bar{X}$ ±S)			*F* = 5.57	0.004		*F* = 1.97	0.142
Never	49 (19.29)	38.87 ± 8.00			43.82 ± 6.17		
Occasionally	106 (41.73)	38.12 ± 7.22			44.61 ± 4.74		
Often	99 (38.98)	41.42 ± 6.90			45.49 ± 4.51		
Insomnia ( $\bar{X}$ ±S)			*t* = −2.29	0.023		*t* = −2.56	0.011
Yes	91 (35.83)	38.14 ± 8.07			43.74 ± 5.47		
No	163 (64.17)	40.34 ± 6.87			45.39 ± 4.60		
Pain ( $\bar{X}$ ±S)			*t* = −0.75	0.452		*t* = −0.41	0.681
No	193 (75.98)	39.35 ± 7.78			44.73 ± 4.84		
Yes	61 (24.02)	40.17 ± 6.00			45.03 ± 5.45		
Cardiac function classification ( $\bar{X}$ ±S)			*F* = 11.97	<0.001		*F* = 2.23	0.109
I class	129 (50.79)	41.27 ± 6.94			45.11 ± 5.15		
II class	102 (40.16)	38.64 ± 7.14			44.87 ± 4.98		
III class	23 (9.06)	33.90 ± 7.62			42.75 ± 3.45		
PCIs ( $\bar{X}$ ±S)			*F* = 0.66	0.575		*F* = 0.88	0.453
0	180 (70.87)	39.51 ± 7.63			44.79 ± 5.09		
1	54 (21.26)	39.09 ± 6.99			44.27 ± 4.67		
2	14 (5.51)	40.13 ± 6.28			46.51 ± 4.47		
3	6 (2.36)	43.48 ± 5.67			46.03 ± 5.44		
Number of medications ( $\bar{X}$ ±S)			*F* = 0.38	0.687		*F* = 1.10	0.333
0	1 (0.39)	45.93			37.52		
1–4	75 (29.53)	39.58 ± 7.78			44.95 ± 4.72		
≥5	178 (70.08)	39.50 ± 7.24			44.78 ± 5.08		
Multimorbidity ( $\bar{X}$ ±S)			*t* = 2.04	0.043		*t* = 0.47	0.638
0–1	203 (79.92)	40.04 ± 6.67			44.88 ± 5.00		
≥2	51 (20.08)	37.59 ± 9.56			44.51 ± 4.95		
Falling ( $\bar{X}$ ±S)			*t* = −0.19	0.851		*t* = 0.47	0.640
No	223 (87.80)	39.52 ± 7.57			44.86 ± 5.05		
Yes	31 (12.20)	39.78 ± 6.00			44.41 ± 4.47		

Continuous variables are expressed as mean ± SD ( $\bar{X}$ ±S) and analyzed by t test or F test; Continuous variables are expressed as the median (interquartile spacing) and analyzed by H test.

### 3.2 Comparison of quality of life scores in different frailty groups

In this study involving 254 participants, the multidimensional frailty prevalence was 53.15% based on the TFI scoring criteria. Frail older patients with coronary artery disease scored lower in physical health (36.62 ± 6.51) compared to their non-frail counterparts (42.88 ± 6.91). Statistically significant differences in physical health were observed between groups with multidimensional frailty, physical frailty, and psychological frailty (*p* < 0.05). Similarly, frailty older patients with CHD scored lower in mental health (43.10 ± 4.75) than non-frailty patients (46.73 ± 4.53). The difference in mental health scores among multidimensional frailty, physical frailty, and psychological frailty groups was also statistically significant (*p* < 0.05). Further details are provided in Table 2.

**Table 2:** Comparison of quality of life scores in different frailty groups (*n* = 254).

Characteristic	Number	PCS	*t*	*p*-value	MCS	*t*	*p*-value
Multidimensional frailty ( $\bar{X}$ ±S)			7.43	<0.001		6.21	<0.001
No	119 (46.85)	42.88 ± 6.91			46.73 ± 4.53		
Yes	135 (53.15)	36.62 ± 6.51			43.10 ± 4.75		
Physical frailty ( $\bar{X}$ ±S)			6.04	<0.001		3.16	<0.001
No	101 (39.76)	42.78 ± 7.12			46.00 ± 5.15		
Yes	153 (60.24)	37.42 ± 6.78			44.01 ± 4.71		
Psychological frailty ( $\bar{X}$ ±S)			4.17	<0.001		4.97	<0.001
No	84 (33.07)	42.21 ± 6.76			46.91 ± 4.55		
Yes	170 (66.93)	38.23 ± 7.34			43.76 ± 4.86		
Social frailty ( $\bar{X}$ ±S)			−0.77	0.440		0.06	0.873
No	233 (91.73)	39.44 ± 7.39			44.82 ± 4.85		
Yes	21 (8.27)	40.74 ± 7.40			44.59 ± 6.34		

PCS, Physical Component Summary; MCS, Mental Component Summary. Continuous variables are expressed as mean ± SD ( $\bar{X}$ ±S) and analyzed by t test.

### 3.3 Examining the relationship between multidimensional frailty and quality of life among older adults with CHD

An examination of the relationship between multidimensional frailty and quality of life in older adults with CHD using Spearman correlation analysis; revealed that multidimensional frailty had a stronger correlation with physical health compared to mental health (*r* = −0.513, *p* < 0.01). Regarding the three components of multidimensional frailty, the PCS showed a moderate negative correlation with physical frailty (*r* = −0.454, *p* < 0.01), while psychological frailty (*r* = −0.360, *p* < 0.01) and social frailty (*r* = −0.228, *p* < 0.01) exhibited weak negative correlations with PCS. The MCS demonstrated moderate negative correlations with physical frailty, psychological frailty, and social frailty (*r* = −0.269, −0.392, −0.265, *p* < 0.01), which were weakly negatively correlated with PCS. Further details can be found in Table 3.

**Table 3:** Examining the relationship between multidimensional frailty and quality of life among older adults with CHD (*n* = 254).

Characteristic	PCS	MCS
Multidimensional frailty	−0.513**	−0.428**
Physical frailty	−0.454**	−0.269**
Psychological frailty	−0.360**	−0.392**
Social frailty	−0.228**	−0.265**

*p-value < 0.05, **p-value < 0.01. PCS, Physical Component Summary; MCS, Mental Component Summary.

### 3.4 Layered regression evaluation of elements influencing the quality of life among older adults with CHD

The stratified regression analysis incorporated variables that demonstrated statistically significant differences in quality of life among older adults with CHD, as identified through univariate analysis. Table 4 presents the results of this analysis. In PCS model 1, age, cardiac function grading, and exercise habits emerged as the primary predictors of PCS in older adults with CHD (*R*^2^ = 0177, *F* = 10.669, *p* < 0.001). PCS model 2, which included multidimensional frailty, revealed that age, cardiac function classification, exercise habits, and multidimensional frailty were the main predictors of quality of life in stroke patients (*R*^2^ = 0.268, *F* = 15.036, Δ*R*^2^ = 0.091, *p* < 0.001). This indicates that multidimensional frailty independently accounts for 9.1% of PCS variance, suggesting a significant negative impact on PCS. For MCS, model 1 identified sleep disturbance as the primary predictor in older adults with CHD (*R*^2^ = 0.025, *F* = 6.554, *p* = 0.011). The addition of multidimensional frailty in MCS model 2 showed that it was the main predictor of MCS (*R*^2^ = 0.138, *F* = 20.102, Δ*R*^2^ = 0.113, *p* < 0.001), independently explaining 11.3% of MCS variance and implying a significant adverse effect on MCS.

**Table 4:** Stratified regression analysis examining factors associated with quality of life among older adults with CHD (*n* = 254).

Model	Independent variables	*β*	SE	*β*′	*t*	*p*-value
PCS						
Model 1	Age	−3.199	0.957	−0.197	−3.578	0.001
	Heart Function Classification	−2.603	0.671	−0.230	−3.881	<0.001
	Exercise	1.696	0.477	0.207	3.553	<0.001
	Insomnia	1.102	0.903	0.072	1.221	0.072
	Multimorbidity	−1.426	0.867	−0.096	−1.644	0.102
Model 2	Age	−2.161	0.924	−0.133	−2.337	0.020
	Heart Function Classification	−2.081	0.641	−0.184	−3.247	0.001
	Exercise	1.262	0.458	0.154	2.755	0.006
	Insomnia	0.158	0.871	0.010	0.181	0.857
	Multimorbidity	−1.473	0.820	−0.100	−1.797	0.074
	Multidimensional frailty	−4.792	0.874	−0.324	−5.481	<0.001
MCS						
Model 1	Insomnia	1.650	0.645	0.159	2.560	0.011
Model 2	Insomnia	0.787	0.626	0.076	1.257	0.210
	Multidimensional frailty	−3.445	0.601	−0.346	−5.729	<0.001

PCS, Physical Component Summary; MCS, Mental Component Summary. Model 1 in PCS: *R*^2^ = 0.177, adjusted *R*^2^ = 0.160, *F* = 10.669, *p* < 0.001; Model 2: *R*^2^ = 0.268, adjusted *R*^2^ = 0.250, *F* = 15.036, Δ*R*^2^ = 0.091, *p* < 0.001; Model 1 in MCS: *R*^2^ = 0.025, adjusted: *R*^2^ = 0.021, *F* = 6.554, *p* = 0.011; Model 2: *R*^2^ = 0.138, adjusted *R*^2^ = 0.131, *F* = 20.102, Δ*R*^2^ = 0.113, *p* < 0.001.

## 4 Discussion

A cross-sectional investigation was conducted to explore the relationship between multifaceted frailty and life quality among older adults with CHD. The study evaluated multidimensional frailty and quality of life in 254 older adults with coronary artery disease. Results indicated that 53.15% of these older adults exhibited multidimensional frailty, with frail individuals scoring lower on the quality of life measures compared to their non-frail counterparts. The research revealed a significant negative correlation between quality of life and multidimensional frailty across physical, psychological, and social domains. Furthermore, stratified linear regression analysis identified multidimensional debility as a key predictor of life quality in this older population.

Contrary to earlier findings, this investigation revealed a substantially higher prevalence of multidimensional frailty in older adults with CHD admitted (53.15% compared to 30.9%) (Liu et al., 2024). The observed difference in frailty prevalence may stem from the study’s use of an integrated frailty model. Unlike traditional frailty assessments, which predominantly focus on physical aspects such as muscle weakness and fatigue, the integrated model expands its scope to incorporate psychological and social frailty. This comprehensive approach likely captures a broader spectrum of frailty-related factors, which may account for the higher prevalence observed in this study. Additionally, variations in measurement tools and criteria across studies can lead to discrepancies in findings. For instance, different frailty indices or scales employed in various studies may influence how frailty is identified and classified, further contributing to the observed differences. The pathogenesis of CHD may also contribute to this difference, as chronic inflammation and immune activation, along with elevated inflammatory markers, contribute to physical debilitation. Additionally, cellular changes, including mitochondrial dysfunction, and metabolic imbalances such as low testosterone and vitamin D deficiency, play a role in somatic deterioration (Veronese et al., 2021). Regarding disease characteristics, older CHD patients typically present with multi-branch lesions and atypical clinical symptoms. These may manifest as asymptomatic myocardial ischemia, ischemic heart failure, or sudden cardiac death (Huayong, 2014). The disease has a prolonged onset and duration, with a 70.3% incidence rate in autumn and winter (Ping and Caihua, 2008) and a recurrence rate of 40–50% (Galimzhanov et al., 2023). From a psychological perspective, these patients experience increased distress due to recurring episodes and financial burdens, leading to higher rates of anxiety and depression. This stress activates the hypothalamic–pituitary–adrenal (HPA) axis, leading to elevated levels of cortisol and adrenaline (James et al., 2023). Chronically elevated hormones can impair cognitive function and increase the incidence of depression (Dziurkowska and Wesolowski, 2021). Research confirms that chronic stress and associated hormonal changes are linked to neurodegenerative diseases and mental health disorders (Knezevic et al., 2023). In addition, lack of social support can cause emotional agitation, accompanied by increased fatigue, headaches, and lethargy (Wang, 2022; Ling et al., 2021), exacerbating the risk of multidimensional frailty. The integrated frailty model suggests that frailty can be reversed or delayed. And multidimensional frailty is prevalent in older patients with CHD, therefore, it is clinically necessary to assess and monitor changes in the condition of older adults with CHD, as well as evaluate and manage frailty holistically and promptly.

This research classified multidimensional frailty into physical, psychological, and social components, revealing that these aspects are linked to the quality of life in older CHD patients during hospitalization. Research has shown that quality of life scores for older CHD patients with comorbid frailty are lower than those without frailty. This aligns with previous studies indicating that frailty worsens the health of these patients, resulting in a diminished quality of life (Veronese et al., 2022; Nguyen and Arnold, 2023). A negative relationship was observed between physical frailty and quality of life (*r* = −0.454, *p* < 0.01); increased physical frailty corresponded with decreased quality of life in older CHD patients. Physical frailty encompasses physiological, endurance, balance, and mobility aspects (Gobbens et al., 2021), and prior research has shown that older CHD patients experience reduced exercise capacity and insufficient physical activity as they age, resulting in diminished quality of life (Katsi et al., 2021; Wardoku et al., 2019). Psychological frailty was negatively associated with quality of life (*r* = −0.454, *p* < 0.01), Nonka et al. (2021) demonstrated that elevated levels of depression and anxiety, coupled with poor social functioning, lead to reduced quality of life and survival rates in CHD patients. Frailty and depression significantly overlap, representing two prevalent conditions in the older cardiovascular population, with pathogenesis linked to inflammation, metabolic dysregulation, and cerebral white matter lesion sites (Aihti et al., 2023). Depression and anxiety are also elements of psychological vulnerability. Similarly, social frailty negatively correlates with quality of life (*r* = −0.454, *p* < 0.01). A longitudinal study emphasized the importance of assessing loneliness, confirming that it predicts quality of life in coronary artery disease patients after 9 months (Fan et al., 2023). This is associated with the repetitive stress of life events and social isolation triggers dysregulation of the sympathetic nerves. The hypothalamic–pituitary–adrenal (HPA) axis may increase inflammation, endothelial dysfunction, atherosclerosis, and a heightened risk of CHD (Gronewold et al., 2021). Recent studies continue to emphasize the complex relationship between social frailty and CHD. One prospective cohort study found that patients with high social isolation had a 15% higher risk of developing CHD compared to those with low social isolation (HR = 1.15, 95% CI: 1.05–1.26; *p* = 0.0017) (Sharif-Nia et al., 2025). Likewise, loneliness was linked to a 15% increased risk of coronary heart disease (HR = 1.15, 95% CI: 1.10–1.20; *p* < 0.001) (Yang et al., 2025). Notably, the correlation coefficient between multidimensional frailty and quality of life surpassed the coefficients for individual physical, mental, and social frailty (*r* = −0.454, *p* < 0.01). This suggests that healthcare professionals should focus on the role of multidimensional frailty in the quality of life of older CHD patients and conduct comprehensive frailty assessments.

Although current research has focussed on physical frailty in older persons with CHD, the present study highlights the importance of incorporating multidimensional physical frailty as a key factor affecting quality of life. This study is unique in that it emphasizes the multidimensional nature of frailty (including physical, psychological and social), which is a departure from previous studies that have focused primarily on physical aspects. This research approach provides a more comprehensive understanding of frailty and its impact on quality of life in older patients with CHD. In addition to this, this study advocates a comprehensive assessment of frailty and personalized interventions based on individual frailty in order to improve the quality of life of older persons with CHD.

Based on the results of this study, we encourage healthcare professionals to conduct regular multidimensional frailty assessments in older patients with coronary artery disease. Such assessments should include physical, psychological, and social aspects to provide a comprehensive assessment of frailty. In addition, comprehensive programmes addressing these dimensions should be designed and implemented to improve the patient’s health. These programmes may include physical interventions: Exercise rehabilitation programmes tailored to the individual’s frailty condition, focusing on improving strength, endurance and mobility. Psychological support: Counseling and mental health interventions to address anxiety, depression and emotional distress. Social support: Community-based programmes and social engagement activities to reduce isolation and improve overall quality of life.

This research has several constraints. First, its cross-sectional nature prevents the establishment of a causal link between multidimensional frailty and quality of life among older individuals with CHD. Second, the study’s limited scope, focusing only on Wuxi, may compromise its accuracy and fail to represent a wider population. To address these limitations, future research should aim to increase the sample size and conduct studies across various locations, thereby enhancing generalizability. Additionally, longitudinal studies are necessary to gain deeper insights into how multidimensional frailty affects the quality of life and prognosis of older patients with CHD following their discharge from the hospital.

## 5 Conclusion

Older adults with CHD frequently experience multidimensional frailty, which is strongly linked to their quality of life. As a key factor influencing life quality, multidimensional frailty should be identified early through screening and assessment by healthcare professionals. To enhance patients’ quality of life and potentially prevent or slow the development of multidimensional frailty, medical staff should implement individualized care strategies.

## Data Availability

The datasets presented in this article are not readily available because of the privacy of the participants. Requests to access the datasets should be directed to yanxiao1109@163.com.

## References

[ref1] Abdullah B. Wolbring G. (2013). Analysis of newspaper coverage of active aging through the Lens of the 2002 World Health Organization active ageing report: a policy framework and the 2010 Toronto charter for physical activity: a global call for action. Int. J. Environ. Res. Public Health 10, 6799–6819. doi: 10.3390/ijerph10126799, PMID: 24317386 PMC3881142

[ref2] Aihti N. Fang S. Ming X. Simin C. Kuribanjiang K. Lei Y. (2023). Progress in the study of the correlation between debility and depression in elderly patients with cardiovascular disease. Nurs. Res. 37, 3487–3491.

[ref3] Byrne R. A. Rossello X. Coughlan J. J. Barbato E. Berry C. Chieffo A.. (2023). 2023 ESC guidelines for the management of acute coronary syndromes. Eur. Heart J. 44, 3720–3826. doi: 10.1093/eurheartj/ehad191, PMID: 37622654

[ref4] Cella A. Veronese N. Pomata M. Leslie Quispe Guerrero K. Musacchio C. Senesi B.. (2021). Multidimensional frailty predicts mortality better than physical frailty in community-dwelling older people: a five-year longitudinal cohort study. Int. J. Environ. Res. Public Health 18:12435. doi: 10.3390/ijerph182312435, PMID: 34886161 PMC8657374

[ref5] Choi J.-Y. Rajaguru V. Shin J. Kim K.-i. (2023). Comprehensive geriatric assessment and multidisciplinary team interventions for hospitalized older adults: a scoping review. Arch. Gerontol. Geriatr. 104:104831. doi: 10.1016/j.archger.2022.104831, PMID: 36279806

[ref6] Chu L. W. Jun W. Tao L. Y. Huanhuan H. Mingzhao X. Qinghua Z. (2019). Analysis of factors affecting quality of life in patients with coronary heart disease based on structural equation modeling. J. Nurs. 34, 20–23.

[ref7] Dent E. Martin F. C. Bergman H. Woo J. Romero-Ortuno R. Walston J. D. (2019). Management of frailty: opportunities, challenges, and future directions. Lancet 394, 1376–1386. doi: 10.1016/s0140-6736(19)31785-4, PMID: 31609229

[ref8] Diseases NCfC, Health TWCotRoC, China Di (2024). Summary of China cardiovascular health and disease report 2023. China Circulation Magazine. 39, 625–660.

[ref9] Dziurkowska E. Wesolowski M. (2021). Cortisol as a biomarker of mental disorder severity. J. Clin. Med. 10:5204. doi: 10.3390/jcm10215204, PMID: 34768724 PMC8584322

[ref10] Fan Y. Ho M. R. Shen B. J. (2023). Loneliness predicts physical and mental health-related quality of life over 9 months among patients with coronary heart disease. Appl. Psychol. Health Well Being 15, 152–171. doi: 10.1111/aphw.12403, PMID: 36184794

[ref11] Galimzhanov A. Sabitov Y. Guclu E. Tenekecioglu E. Mamas M. A. (2023). Phenotyping for percutaneous coronary intervention and long-term recurrent weighted outcomes. Int. J. Cardiol. 374, 12–19. doi: 10.1016/j.ijcard.2022.12.035, PMID: 36574846

[ref12] Gobbens R. J. Krans A. van Assen M. A. (2015). Validation of an integral conceptual model of frailty in older residents of assisted living facilities. Arch. Gerontol. Geriatr. 61, 400–410. doi: 10.1016/j.archger.2015.06.001, PMID: 26293001

[ref13] Gobbens R. J. J. van Assen M. Augustijn H. Goumans M. van der Ploeg T. (2021). Prediction of mortality by the Tilburg frailty Indicator (TFI). J. Am. Med. Dir. Assoc. 22, 607.e1–607.e6. doi: 10.1016/j.jamda.2020.07.033 32883597

[ref14] Gronewold J. Engels M. van de Velde S. Cudjoe T. K. M. Duman E.-E. Jokisch M.. (2021). Effects of life events and social isolation on stroke and coronary heart disease. Stroke 52, 735–747. doi: 10.1161/STROKEAHA.120.032070, PMID: 33445957

[ref15] Hongyu W. Z. (2016). Measurement reliability and validity of the short form quality of life scale (SF-12) in rural older adults. J. Shanghai Jiao Tong Univ. 36, 1070–1074.

[ref16] Huayong Y. (2014). A clinical study of the characteristics and risk factors of elderly patients with coronary heart disease. J. Cardiovasc. Rehabil. Med. 23, 256–258.

[ref17] James K. A. Stromin J. I. Steenkamp N. Combrinck M. I. (2023). Understanding the relationships between physiological and psychosocial stress, cortisol and cognition. Front. Endocrinol. 14:1085950. doi: 10.3389/fendo.2023.1085950, PMID: 36950689 PMC10025564

[ref18] Katsi V. Georgiopoulos G. Mitropoulou P. Kontoangelos K. Kollia Z. Tzavara C.. (2021). Exercise tolerance and quality of life in patients with known or suspected coronary artery disease. Qual. Life Res. 30, 2541–2550. doi: 10.1007/s11136-021-02844-y 33893931

[ref19] Knezevic E. Nenic K. Milanovic V. Knezevic N. N. (2023). The role of cortisol in chronic stress, neurodegenerative diseases, and psychological disorders. Cells 12:2726. doi: 10.3390/cells12232726, PMID: 38067154 PMC10706127

[ref20] Ling C. Minhua L. Jinhua G. (2021). Expert consensus on psychological care of hospitalized patients with coronary heart disease. J. Nurs. 28, 45–51. doi: 10.16460/j.issn1008-9969.2021.22.045

[ref21] Liu Y. F. Ding R. J. Meng X. P. Wang L. M. Shen X. Y. Shen L.. (2023). Self-reported quality of life in patients with coronary heart disease and analysis of the associated factors. Zhonghua Nei Ke Za Zhi 62, 384–392. doi: 10.3760/cma.j.cn112138-20220524-00404, PMID: 37032133

[ref22] Liu S. Q. Yuan X. L. Liang H. T. Jiang Z. X. Yang X. L. Gao H. M. (2024). Development and validation of frailty risk prediction model for elderly patients with coronary heart disease. BMC Geriatrics 24:742. doi: 10.1186/s12877-024-05320-7, PMID: 39244543 PMC11380413

[ref23] Maidan M. L. Pei X. Fengmei X. Fenglan W. Pan Z. Xianyao L.. (2023). A study of the effects of active aging and social support on the frailty of community-dwelling older adults. J. Nurs. Manag. 23, 669–673.

[ref24] Miao C. H. Lu L. Fenfen S. Jiaji Y. Shuiping H. (2019). Quality of life and influencing factors before and after coronary intervention in elderly patients with coronary artery disease. Chin. J. Gerontol. 39, 3813–3816.

[ref25] Nguyen D. D. Arnold S. V. (2023). Impact of frailty on disease-specific health status in cardiovascular disease. Heart 109, 977–983. doi: 10.1136/heartjnl-2022-321631, PMID: 36604164 PMC10272004

[ref26] Ning Z. Wenling Z. Xiaohong L. Wei C. Junren K. Minglei Z.. (2021). The effect of debility on the short-term prognosis of hospitalized elderly patients with coronary heart disease: a prospective cohort study. Concordia Med. J. 12, 59–66.

[ref27] Nonka T. G. Lebedeva E. V. Repin A. N. (2021). Influence of depressive disorders on quality of life and three-year survival of patients with coronary heart disease. Eur. Heart J. 42:2752. doi: 10.1093/eurheartj/ehab724.2752

[ref28] Parra-Rizo M. A. (2020). Efecto y adecuación del ejercicio para la mejora cardiovascular de la población mayor de 65 años. Revista de PSICOLOGÍA DE LA SALUD 8:8. doi: 10.21134/pssa.v8i1.670

[ref29] Ping Z. Caihua Z. (2008). Analysis of the correlation between the incidence of cardiovascular events and seasonal changes in geriatric patients and anticipatory care. Nurs. Res. 2, 121–122.

[ref30] Ping N. Jingli C. Na L. (2010). Sample size estimation for quantitative studies in nursing research. Chin. J. Nurs. 45, 378–380.

[ref31] Rivera Miranda P. Trujillo Altamirano C. Yáñez-Yáñez R. NMA D. Quintana-Peña P. Parra-Rizo M. A. (2024). Entrenamiento de fuerza para prevención de caídas en personas mayores: Una revisión sistemática. Revista Salud Uninorte 40, 216–238. doi: 10.14482/sun.40.01.650.452

[ref32] Sharif-Nia H. Jackson A. C. Salehi S. Miraghai F. Hosseini S. H. (2025). Loneliness and repetitive negative thinking mediate the link between social health and cardiac distress in heart disease patients. Sci. Rep. 15:11804. doi: 10.1038/s41598-025-96968-7, PMID: 40189696 PMC11973143

[ref33] Shou J. Ren L. Wang H. Yan F. Cao X. Wang H.. (2016). Reliability and validity of 12-item short-form health survey (SF-12) for the health status of Chinese community elderly population in Xujiahui district of Shanghai. Aging Clin. Exp. Res. 28, 339–346. doi: 10.1007/s40520-015-0401-9, PMID: 26142623

[ref34] Tang M. Wang S. H. Li H. L. Chen H. Sun X. Y. Bian W. W.. (2021). Mental health status and quality of life in elderly patients with coronary heart disease. PeerJ 9:e10903. doi: 10.7717/peerj.10903, PMID: 33643714 PMC7896500

[ref35] Veronese N. Koyanagi A. Smith L. Musacchio C. Cammalleri L. Barbagallo M.. (2021). Multidimensional frailty increases cardiovascular risk in older people: an 8-year longitudinal cohort study in the osteoarthritis initiative. Exp. Gerontol. 147:111265. doi: 10.1016/j.exger.2021.111265, PMID: 33539985

[ref36] Veronese N. Noale M. Cella A. Custodero C. Smith L. Barbagelata M.. (2022). Multidimensional frailty and quality of life: data from the English longitudinal study of ageing. Qual. Life Res. 31, 2985–2993. doi: 10.1007/s11136-022-03152-9, PMID: 35579730 PMC9470717

[ref37] Wang Z. (2022). Effects of multidimensional evidence-based nursing on psychological status and quality of life of elderly patients with coronary heart disease. Modern J. Integr. Chinese Western Med. 31, 2603–2606.

[ref38] Wang Z. W. (2023). Status of cardiovascular disease in China. J. Geriatr. Cardiol. 20, 397–398. doi: 10.26599/1671-5411.2023.06.006, PMID: 37416520 PMC10320770

[ref39] Wardoku R. Blair C. Demmer R. Prizment A. (2019). Association between physical inactivity and health-related quality of life in adults with coronary heart disease. Maturitas 128, 36–42. doi: 10.1016/j.maturitas.2019.07.005, PMID: 31561820 PMC7261413

[ref40] Ware J. Jr. Kosinski M. Keller S. D. (1996). A 12-item short-form health survey: construction of scales and preliminary tests of reliability and validity. Med. Care 34, 220–233. doi: 10.1097/00005650-199603000-00003, PMID: 8628042

[ref41] Xiamin P. Feng L. Yaxi T. Houqiang H. Yongli H. Shengmin G. (2023). Meta-analysis of the incidence of debility and factors affecting it in elderly patients with coronary heart disease. J. Continuing Nurs. Educ. 38, 1012–6+42. doi: 10.16821/j.cnki.hsjx.2023.25.009

[ref42] Xing X. Guifang G. Jing S. (2013). A reliability study of the Chinese version of the Tilburg frailty assessment scale (TFAS). J. Nurs. 20, 1–5. doi: 10.16460/j.issn1008-9969.2013.16.006

[ref43] Xue R. Chen B. Y. Ma R. H. Zhang Y. X. Zhang K. L. (2024). Association of multidimensional frailty and quality of life in middle-aged and older people with stroke: a cross-sectional study. J. Clin. Nurs. 33, 1562–1570. doi: 10.1111/jocn.16969, PMID: 38131358

[ref44] Yang Z. Liu C. He L. Zhao H. Jian J. Chen H.. (2025). Social isolation and loneliness increase the risk of coronary heart disease: insights from a prospective cohort study. Soc. Sci. Med. 366:117701. doi: 10.1016/j.socscimed.2025.117701, PMID: 39842386

[ref45] Yuhui C. Qingqing L. Mei W. Meng X. (2024). Current status of the development and application of a conceptual framework-based debilitation assessment tool. J. Continuing Nurs. Educ. 39, 1113–1117. doi: 10.16821/j.cnki.hsjx.2024.10.019

[ref46] Zhou Q. Li Y. Gao Q. Yuan H. Sun L. Xi H.. (2023). Prevalence of frailty among Chinese community-dwelling older adults: a systematic review and meta-analysis. Int. J. Public Health 68:1605964. doi: 10.3389/ijph.2023.1605964, PMID: 37588041 PMC10425593

